# Design and Evaluation of an Immersive, Interactive Acceptance and Commitment Therapy (ACT) Intervention Targeting OCD, Depression, and Anxiety in Medical Students: A Pilot Study

**DOI:** 10.1192/bjo.2026.11057

**Published:** 2026-06-30

**Authors:** Athanasios Hassoulas, Noor Al-Helu, Zahra Jaffery, Erica Tuma, Iarl Sapong

**Affiliations:** Cardiff University, Cardiff, United Kingdom

## Abstract

**Aims:**

Acceptance and Commitment Therapy (ACT) is a third-wave cognitive-behavioural therapeutic modality that promotes psychological flexibility through six core principles: acceptance, cognitive defusion, contact with the present moment, self-as-context, values, and committed action. This pilot study aimed to evaluate the effectiveness of an immersive ACT intervention delivered in a fully interactive environment for reducing symptoms of depression, anxiety, and obsessive-compulsive disorder (OCD) among medical students. The immersive ACT experience was created by uploading and adapting ACT therapeutic content into the Immersive Studio software, which projected the material onto the walls of the suite. Participants could interact with the content presented visually and auditorily by touching the walls. The use of immersive technology was intended to enhance engagement and experiential learning of ACT principles, thereby reducing symptoms of depression, anxiety, and/or OCD.

**Methods:**

A pre-post intervention design was implemented at Cardiff University’s School of Medicine HIVE immersive learning suite: a 5×5 metre room equipped with fully immersiveand interactive capabilities. This environment enabled participants to engage with ACT content across three immersive modules, each designed to teach and apply ACT’s six core principles in a dynamic and experiential manner. Pre-and post-intervention scores were collated using the Patient Health Questionnaire-9 (PHQ-9) for depression, Generalised Anxiety Disorder-7 (GAD-7) for anxiety, and Obsessive-Compulsive Inventory-Revised (OCI-R) for OCD as standardised, validated measures.

**Results:**

Ten medical students completed all elements of the three immersive ACT modules, as well as the pre and post measures. Scores from the three validated measures were analysed using the Wilcoxon Signed Rank test to assess changes in symptom severity. Depressive symptoms significantly decreased following the intervention, with mean PHQ-9 scores reducing from 7.13 to 4.00 (z=−2.207, p <0.05). No statistically significant changes were observed in anxiety (GAD-7) or OCD (OCI-R) scores.

**Conclusion:**

Delivering ACT as an immersive and interactive therapeutic modality appears to be an effective approach for reducing depressive symptoms among medical students. This pilot study highlights the potential of immersive environments to enhance psychological interventions and enhance psychological flexibility. Further research with larger samples (currently underway) is required to examine its impact on anxiety, depression and OCD symptoms and to optimise immersive delivery for broader mental health benefits.

